# Nanoscale on-chip all-optical logic parity checker in integrated plasmonic circuits in optical communication range

**DOI:** 10.1038/srep24433

**Published:** 2016-04-13

**Authors:** Feifan Wang, Zibo Gong, Xiaoyong Hu, Xiaoyu Yang, Hong Yang, Qihuang Gong

**Affiliations:** 1 State Key Laboratory for Mesoscopic Physics & Department of Physics, Collaborative Innovation Center of Quantum Matter, Peking University, Beijing 100871, People’s Republic of China; 2 Collaborative Innovation Center of Extreme Optics, Shanxi University, Taiyuan 030006, People’s Republic of China

## Abstract

The nanoscale chip-integrated all-optical logic parity checker is an essential core component for optical computing systems and ultrahigh-speed ultrawide-band information processing chips. Unfortunately, little experimental progress has been made in development of these devices to date because of material bottleneck limitations and a lack of effective realization mechanisms. Here, we report a simple and efficient strategy for direct realization of nanoscale chip-integrated all-optical logic parity checkers in integrated plasmonic circuits in the optical communication range. The proposed parity checker consists of two-level cascaded exclusive-OR (XOR) logic gates that are realized based on the linear interference of surface plasmon polaritons propagating in the plasmonic waveguides. The parity of the number of logic 1s in the incident four-bit logic signals is determined, and the output signal is given the logic state 0 for even parity (and 1 for odd parity). Compared with previous reports, the overall device feature size is reduced by more than two orders of magnitude, while ultralow energy consumption is maintained. This work raises the possibility of realization of large-scale integrated information processing chips based on integrated plasmonic circuits, and also provides a way to overcome the intrinsic limitations of serious surface plasmon polariton losses for on-chip integration applications.

Optical computing, where photons are used as information carriers, has the potential to realize ultrahigh-speed ultrawide-band information processing. An all-optical logic parity checker has the function of determining the parity of the number of logic 1 signals propagating in optical computing systems and thus determining whether the logic signals are correctly transmitted, i.e., the logic signals are correct for an even number of logic 1s, while an error exists in the logic signals for an odd number of logic 1s^1^. The nanoscale chip-integrated all-optical logic parity checker is an indispensable core component of optical computing systems. The all-optical logic parity checker must have several essential properties to be suitable for practical applications, including ultrasmall feature size, ultralow energy consumption, a high-intensity contrast ratio between the logic states 1 and 0, and on-chip operation capability. From a theoretical perspective, various schemes have been proposed for demonstration of the all-optical logic parity checking function, including use of the electro-optic effect in lithium niobate crystals^1^, semiconductor optical amplifiers^2^,^3^,^4^,^5^, third-order nonlinear optical materials^6^,^7^,^8^, and spatial light modulators^9^. From an experimental perspective, Poustie *et al.* reported an all-optical parity checker with bit-differential delay that used semiconductor optical amplifiers with an operating threshold signal light intensity of the GW/cm^2^ order^10^. Because of the relatively low third-order nonlinear susceptibilities of both conventional semiconductors and organic polymers, a high threshold signal light intensity of the GW/cm^2^ order is required to trigger the operation of all-optical parity checkers based on either semiconductor optical amplifiers or third-order nonlinear optical effects^10^. The large sizes of both electro-optic crystals and spatial light modulators, which are of the order of several millimeters, do not meet the on-chip integration compatibility requirements. In addition, no effective mechanism for the realization and fabrication of nanoscale chip-integrated all-optical logic parity checkers has been available to date. Little experimental progress has thus been made in practical device development because of material bottleneck limitations and the lack of effective realization mechanisms^10^. This has greatly restricted the practical application of nanoscale chip-integrated all-optical logic parity checkers.

Here, we report a simple and effective strategy for direct realization of a nanoscale chip-integrated all-optical logic parity checker with ultracompact feature size, ultralow energy consumption, and high contrast ratio in an integrated plasmonic circuit. The all-optical logic parity checker consists of two-level cascaded exclusive-OR (XOR) logic gates, which are realized on the basis of the linear interference of surface plasmon polaritons (SPPs) propagating in U-shaped plasmonic waveguides. When compared with conventional plasmonic slot waveguides, the distinctive features of the U-shaped plasmonic waveguides lie in their strong light-field localization, relatively low propagation losses, high transmission around sharp bends, and high tolerance to structural imperfections^11^,^12^, as described in detail in the Supplementary Information. Additionally, the intrinsic ohmic losses of gold and silver in the optical communication range are much smaller than those in the visible and near-infrared ranges, which means that SPP modes have much longer propagation lengths in U-shaped plasmonic waveguides^13^. Therefore, the major obstacles that limit the on-chip integration applications of SPPs, i.e., the serious ohmic losses of gold and silver and the resulting short propagation lengths of the SPPs, can be overcome to a certain degree by U-shaped plasmonic waveguides operating in the optical communication range. The U-shaped plasmonic waveguide can confine the signal light to the subwavelength-scale, thus resulting in ultralow feature sizes for the all-optical logic parity checker. Because the parity checking function is realized on the basis of the linear interference of SPPs, the all-optical logic parity checker does not have any high operating power requirements, which thus ensures ultralow energy consumption. The precisely controlled optical phase differences between different U-shaped plasmonic waveguides and the quasi-monochromatic SPP modes that can be excited by a continuous-wave (CW) laser beam operating at a wavelength of 1560 nm ensure a high intensity contrast ratio of 30 dB between the output logic states of 1 and 0. The parity checker function is performed using the U-shaped plasmonic waveguides, i.e., it is performed in the direction parallel to the plasmonic circuit surface, which guarantees the required on-chip operation characteristics. In our experiments, the incident signal light intensity was only 100 kW/cm^2^, which is four orders of magnitude lower than that of previously reported devices based on semiconductor optical amplifiers and third-order nonlinear optical materials^2^,^3^,^4^,^5^,^6^,^7^,^8^,^10^, while the overall feature size is reduced by more than two orders of magnitude and ultralow energy consumption is maintained. This work not only raises the possibility of realization of large-scale integrated information processing chips based on integrated plasmonic circuits, but also offers a way to overcome the intrinsic limitations of the serious losses of SPPs for on-chip integration applications.

## Results

### Waveguiding properties of U-shaped plasmonic waveguides

**Figure 1:**
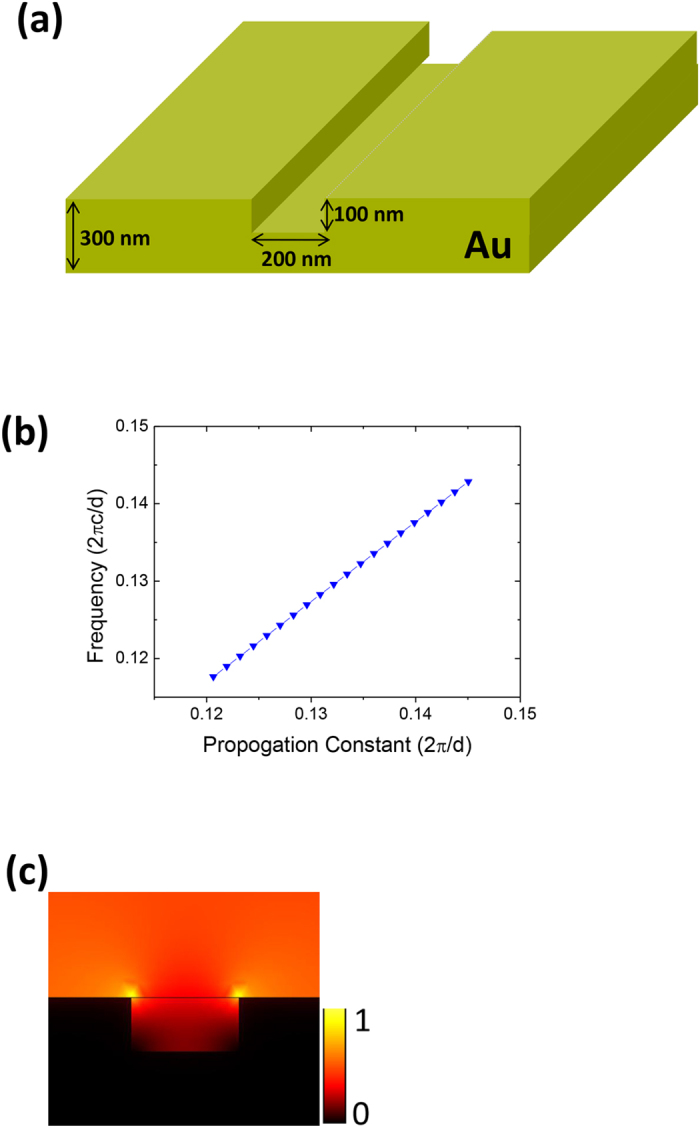
Characterization of the U-shaped plasmonic waveguide. (**a**) Schematic diagram of waveguide structure. (**b**) Dispersion relations. (**c**) Power density profile of guided mode excited by incident CW light at a wavelength of 1560 nm.

The U-shaped plasmonic waveguide structure is shown schematically in Fig. 1(a). The U-shaped plasmonic waveguide consists of an infinitely-long air groove with width of 200 nm and depth of 100 nm etched in a 300-nm-thick gold film. The dispersion relations of the plasmonic waveguide were calculated by the finite element method (using the commercial software package COMSOL Multiphysics)^14^, and the calculated results are shown in Fig. 1(b). The refractive index of air was set to 1, and the wavelength-dependent complex refractive index of gold was obtained from ref. 15. The U-shaped plasmonic waveguide can offer wideband guided SPP modes, as confirmed by the calculations of Li *et al.*^16^. To further confirm the properties of these guided SPP modes, we calculated the power density profile of a guided mode excited by continuous wave (CW) incident light at a wavelength of 1560 nm by the finite element method, and the calculated results are shown in Fig. 1(c). The guided mode is mainly confined within the groove region but extends slightly into the adjacent air regions. The waveguide is not etched completely through the gold film, although the energy would be confined to a smaller extent for reasons that will be stated at the end of this section. The maximum intensity is located at the gold-air interface around the two vertexes on the upper side, which is consistent with the calculations of Li *et al.*^16^. In our experiment, we etched a coupling grating connected to an air groove using a triangular configuration at the input port of the U-shaped plasmonic waveguide for efficient excitation and collection of the required SPPs for an input logic signal of 1. The grating period, the air groove length, the air groove width, and the air groove depth of this input coupling grating were 1.12 μm, 3 μm, 560 nm, and 300 nm, respectively. The input coupling grating was etched through the gold film to realize effective conversion from the incident light in free space to the SPP modes. The lateral length and depth of the triangular air groove were 3 μm and 100 nm, respectively. We also etched a grating into the output port to couple the guided SPP modes into free space for measurement purposes. The grating period, the air groove length, the air groove width, and the air groove depth of this decoupling grating were 1.12 μm, 3 μm, 560 nm, and 100 nm, respectively. Neither the U-shaped plasmonic waveguide nor the decoupling grating were etched through the gold film, thus ensuring that only the required scattering light signal of the SPP mode in the output waveguide can be obtained from the decoupling grating.

### All-optical logic XOR gate performance

**Figure 2:**
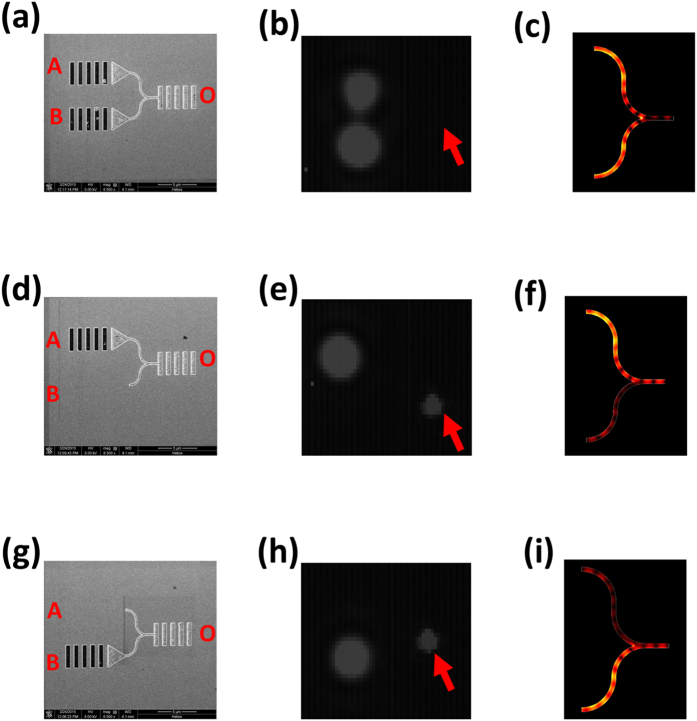
Logic operation of all-optical XOR gate. (**a**) SEM image of the sample, (**b**) CCD image measured under 1560 nm CW laser excitation, and (**c**) simulated electric field distribution for a 1560 nm CW incident laser beam for the logic operation “1 XOR 1 = 0”. (**d**) SEM image of the sample, (**e**) measured CCD image under excitation of a 1560 nm CW laser, and (**f**) simulated electric field distribution results for a 1560 nm CW incident laser beam for the logic operation “1 XOR 0 = 1”. (**g**) SEM image of the sample, (**h**) measured CCD image under 1560 nm CW laser excitation , and (**i**) simulated electric field distribution results for a 1560 nm CW incident laser for the logic operation “0 XOR 1 = 1”. The arrow indicates the position of the decoupling grating of output waveguide O.

The all-optical logic XOR gate has an asymmetric Y-shaped configuration, as shown in Fig. 2(a), with a bending region that has a circular arc shape with a radius of 1.5 μm to reduce the propagation losses^17^. According to our finite element method-based calculations, a guided SPP mode with a wavelength of 1100 nm can be excited in the U-shaped plasmonic waveguide by an input 1560 nm CW signal laser beam. There is an optical path difference of 550 nm between the U-shaped plasmonic waveguides A and B, which indicates that destructive interference can be produced in the output waveguide for the guided SPP modes that are propagating in plasmonic waveguides A and B under 1560 nm signal laser beam excitation. To perform the logic operation “1 XOR 1 = 0”, we etched two coupling gratings that were connected via triangular air grooves in the input ports of plasmonic waveguides A and B, which means that the SPP modes can be excited in plasmonic waveguides A and B simultaneously. A decoupling grating was also etched into the output port of waveguide O to couple the guided SPP modes into free space to be measured. The charge-coupled device (CCD) image measured under the excitation of a 1560 nm CW laser is shown in Fig. 2(b). A very weak signal is output from the decoupling grating with an intensity of 0.007 a.u. because destructive interference was produced in the output waveguide. This corresponds to the output logic state 0. Therefore, the logic operation of “1 XOR 1 = 0” was realized. The simulated electric field distribution when the SPP mode is incident in both plasmonic waveguides A and B is shown in Fig. 2(c). No signal can be obtained from the output waveguide, thus confirming the logic operation “1 XOR 1 = 0”. To perform this logic operation, we etched a coupling grating that was connected via triangular air grooves only to the input port of plasmonic waveguide A, as shown in Fig. 2(d). This means that the SPP mode can only be excited in plasmonic waveguide A, and no SPP mode can thus be excited in plasmonic waveguide B. The measured CCD image under 1560 nm CW laser excitation is shown in Fig. 2(e). A strong signal from the decoupling grating with intensity of 45 a.u. was obtained at the output port. This corresponds to the output logic state of 1. Therefore, the logic operation “1 XOR 0 = 1” was realized. The simulated electric field distribution when the SPP mode is incident in plasmonic waveguide A is shown in Fig. 2(f). A strong signal can be obtained from the output waveguide, thus confirming the logic operation “1 XOR 0 = 1”. Similarly, when we etched a coupling grating connected using triangular air grooves in the input port of plasmonic waveguide B only, as shown in Fig. 2(g), the logic operation “0 XOR 1 = 1” (Fig. 2[h]) was realized under 1560 nm CW laser excitation. The simulated electric field distribution (Fig. 2[i]) when the SPP mode is incident in plasmonic waveguide B indicates that a strong signal can be obtained from the output waveguide. This confirms that the logic operation “0 XOR 1 = 1” is performed. Liu *et al.* showed that the intensity contrast ratio between the output logic values of 1 and 0 was calculated from 10·log(*P1*/*P0*), where *P1* and *P0* are the signal intensities of the logic 1 and logic 0, respectively^18^. The measured intensity contrast ratio between the output logic 1 and logic 0 reached 38.1 dB.

### All-optical logic parity checking performance

**Figure 3:**
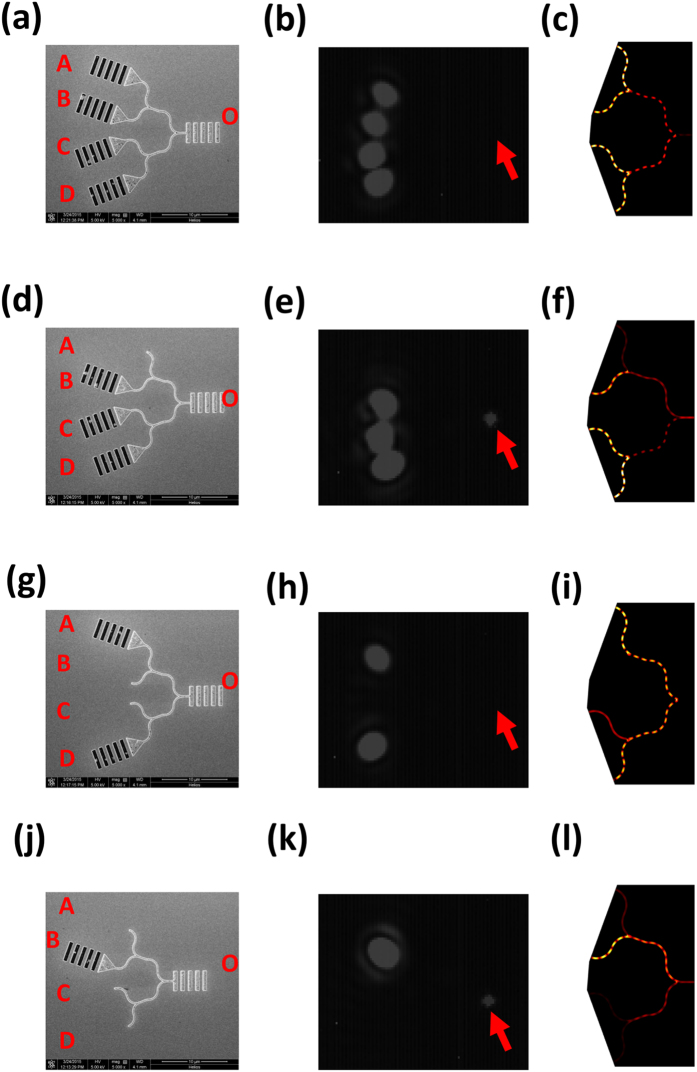
Logic operation of all-optical logic parity checker. (**a**) SEM image of the sample, (**b**) measured CCD image under 1560 nm CW laser excitation, and (**c**) simulated electric field distribution results for a 1560 nm CW incident laser for incident logic signal 1111. (**d**) SEM image of the sample, (**e**) CCD image measured under 1560 nm CW laser excitation, and (**f**) simulated electric field distribution results for a 1560 nm CW incident laser for the incident logic signal 0111. (**g**) SEM image of the sample, (**h**) measured CCD image under 1560 nm CW laser excitation, and (**i**) simulated electric field distribution results for a 1560 nm CW incident laser for incident logic signal 1001. (**j**) SEM image of the sample, (**k**) measured CCD image under 1560 nm CW laser excitation, and (**l**) simulated electric field distribution results for a 1560 nm CW incident laser for incident logic signal 0100. The arrow indicates the position of the decoupling grating of output waveguide O.

The sample all-optical logic parity checker consists of two-level cascaded XOR logic gates that were formed using U-shaped plasmonic waveguides, as shown in Fig. 3(a), with a total of four input plasmonic waveguides (marked A, B, C, and D), and three XOR gates. The sample all-optical logic parity checker can be used to discriminate the parity of the number of logic 1s in the incident four-bit logic signals. The highest and lowest logic digits were incident in plasmonic waveguides A and D, respectively. The two middle logic digits were incident in plasmonic waveguides B and C, respectively. There is an optical path difference of 550 nm between the two adjacent U-shaped plasmonic waveguides for each XOR gate, which indicates that destructive interference can be obtained in the output waveguide for guided SPP modes excited by the 1560 nm signal laser beam. To determine the parity of the number of logic 1s for the incident four-bit logic signal 1111, we etched four coupling gratings connected using triangular air grooves into the input ports of plasmonic waveguides A, B, C, and D, which means that the SPP modes can be excited in all four plasmonic waveguides simultaneously. A decoupling grating was also etched into the output port of waveguide O to couple the guided SPP modes into free space to be measured. The measured CCD image under 1560 nm CW laser excitation is shown in Fig. 3(b). A very weak signal is output from the decoupling grating of output waveguide O with an intensity of 0.005 a.u. because destructive interference was achieved in the output waveguides for the two XOR gates on the first level. This corresponds to an output logic state of 0, and indicates that the incident four-bit logic signal 1111 has an even parity of logic 1. The calculated electric field distribution of the all-optical parity checker under 1560 nm CW laser excitation is shown in Fig. 3(c). No scattering signal was obtained from output waveguide O, thus confirming the measured results. To determine the parity of the number of logic 1s for the incident four-bit logic signal 0111, we etched three coupling gratings connected using triangular air grooves into the input ports of plasmonic waveguides B, C, and D, as shown in Fig. 3(d), which means that SPP modes can be excited in plasmonic waveguides B, C, and D simultaneously, but no SPP mode can be excited in plasmonic waveguide A. The measured CCD image under 1560 nm CW laser excitation is shown in Fig. 3(e). There is a strong output signal from the decoupling grating of output waveguide O with intensity of 35 a.u. The outputs of the two XOR gates in the first level were 1 and 0, which means that the second level XOR gate output becomes 1. This indicates that the incident four-bit logic signal 0111 has an odd parity of logic 1. The calculated electric field distribution of the all-optical parity checker under 1560 nm CW laser excitation is shown in Fig. 3(f). A strong scattering signal can be obtained from output waveguide O, which again confirms the measured results. The measured intensity contrast ratio between the output logic 1 and logic 0 reached 38.5 dB. To determine the parity of the number of logic 1s for the incident four-bit logic signal 1001, we etched two coupling gratings connected via triangular air grooves into the input ports of plasmonic waveguides A and D, which means that SPP modes can be excited in plasmonic waveguides A and D simultaneously, and that no SPP modes can be excited in plasmonic waveguides B and C. The measured CCD image under 1560 nm CW laser excitation is shown in Fig. 3(g). A very weak signal is output from the decoupling grating of output waveguide O with an intensity of 0.004 a.u., as shown in Fig. 3(h). XOR gates in the first level had an output of 1, which means that the output of the second level XOR gate becomes 0. This shows that the incident four-bit logic signal 1001 has an even parity of logic 1. The calculated electric field distribution of the all-optical parity checker under 1560 nm CW laser excitation is shown in Fig. 3(i). No scattering signal can be obtained from output waveguide O, thus confirming the measured results. To determine the parity of the number of logic 1s for the incident four-bit logic signal 0100, we etched a single coupling grating connected via triangular air grooves into the input port of plasmonic waveguide B, as shown in Fig. 3(j), which means that the SPP mode can be excited in plasmonic waveguide B only, and no SPP modes can be excited in plasmonic waveguides A, C, and D. The measured CCD image under 1560 nm CW laser excitation is shown in Fig. 3(k). Again, a strong signal is output from the decoupling grating of output waveguide O with an intensity of 32 a.u. The outputs of the two XOR gates in the first level were logic 1 and logic 0, which means that the output of the second level XOR gate becomes logic 1. Therefore, the incident four-bit logic signal 0100 has an odd parity of logic 1. The calculated electric field distribution of the all-optical parity checker under 1560 nm CW laser excitation is shown in Fig. 3(l). A strong scattering signal can be obtained from output waveguide O, again confirming the measured results.

## Discussion

Because of the strong field confinement effect of U-shaped plasmonic waveguides, the minimum feature size of the sample all-optical parity checker was only 15 μm, which is more than two orders of magnitude smaller than the previously reported results^1^,^2^,^3^,^4^,^5^,^6^,^7^,^8^,^9^. The mechanism for realization of the all-optical parity checker was based on linear interference of the SPPs, and there are thus no high-power requirements. A low-power CW laser beam can thus trigger the all-optical parity checking operation. The operating intensity of the signal light was as low as 100 kW/cm^2^ in our experiment, and this represents a reduction of four orders of magnitude when compared with the corresponding values from previous reports^1^,^2^,^3^,^4^,^5^,^6^,^7^,^8^,^9^. Nozaki *et al.* noted that the ultralow incident signal light intensity of only 100 kW/cm^2^ is comparable with that of efficient ultralow-power all-optical switching devices based on silicon photonic crystal nanocavities^19^. According to our calculations, the coupling efficiency of the input coupling grating was 30%, corresponding to an insertion loss of 5 dB, which is sufficient to realize high-efficiency conversion from the incident light in free space to the SPP modes. These results have been confirmed by the measurements of Ishi *et al.*^20^. The excellent light-to-SPP conversion efficiency and the superb propagation lengths of the guided SPP modes in the U-shaped plasmonic waveguides also contribute to the ultralow energy consumption of the proposed all-optical logic parity checker devices. In addition, the precisely controlled optical phase difference between the different U-shaped plasmonic waveguides and the quasi-monochromatic SPP modes excited by the CW laser beam guarantee a high-intensity contrast ratio between the output logic states of 1 and 0 of more than 38 dB. Therefore, the all-optical parity checker has the unique characteristics of ultrasmall feature size, ultralow energy consumption, and an ultrahigh contrast ratio, along with a capability for direct on-chip operation in plasmonic circuits. For several cascaded plasmonic microstructures, the insertion loss of the input-coupling port that connects two plasmonic microstructures can be increased. According to the results of our experiments and calculations, the intensity contrast ratio between the output logic 1 and the corresponding logic 0 reached 20 dB when the incident light wavelength was changed from 1560 nm to 1552 nm (or 1569 nm). An operating optical bandwidth of 17 nm can thus be obtained for the all-optical logic parity checker.

The output logic state of the sample all-optical parity checker described above not only reflects the parity of the logic 1 in the incident four-bit logic signals, but can also be used as the parity bit for the incident logic signals. The sample all-optical parity checker can therefore also function as an all-optical parity generator for the incident four-bit logic signals. We can add a parity bit, i.e., the output logic state of the all-optical parity checker sample, to the last part of the incident logic signal to form new five-bit logic signals where the lowest digit is used as the parity bit. The strategy proposed here can also easily be extended to realize the functionality of an all-optical checker and generator to be applied to multi-bit complex logic signals by simply increasing the number of XOR gates in the first and second levels. This work therefore not only paves the way towards the construction of ultrahigh-speed and ultrawide-band information processing chips and optical computing systems based on plasmonic nanostructures, but also offers a way to overcome the intrinsic obstacle of the SPPs for ultralarge-scale on-chip integration applications. These results are completely different to the results obtained in ref. 14, as described in detail in the supplementary information. However, the optical path difference between two plasmonic waveguides can be controlled easily and precisely via the microfabrication etching technology. The subwavelength-scale light confinement effect of the plasmonic modes means that the feature sizes of the integrated photonic devices based on plasmonic waveguides can be as compact as those based on semiconductor nanowires. In addition, it would be easier to construct complex all-optical logic devices when using plasmonic waveguides based on third-order nonlinear optical effects.

In an actual data stream, the bit signals appear sequentially. Wood *et al.* noted that a high quality factor of more than 4000 can be obtained in a plasmonic coupled resonator optical waveguide, and this indicates that plasmonic coupled resonator optical waveguides are excellent candidates for construction of the delay line unit for the data stream^21^. Each bit signal from the data stream can initially be coupled into a different delay line unit composed of a plasmonic coupled resonator optical waveguide with different structural parameters, and this guarantees that any bit signal that arrives early is subject to a longer time delay. The output bit signals from these delay line units can then be coupled simultaneously into the all-optical parity checker, which can ensure correct implementation of the parity checking operation for any arbitrary input 4-bit logic signals. Weeber *et al.* noted that plasmonic coupled resonator optical waveguides could also be constructed using coupled plasmonic coplanar nanocavities formed by several defect units in a one-dimensional plasmonic crystal consisting of an array of periodic nanogratings etched at the center of a plasmonic waveguide^22^. Balci *et al.* also stated that this type of plasmonic coupled resonator optical waveguide was highly suitable for integration into a plasmonic platform with a waveguiding geometry^23^.

In summary, we have directly realized a nanoscale chip-integrated all-optical logic parity checker in integrated plasmonic circuits. An overall minimum feature size of 15 μm, ultralow energy consumption, and an ultrahigh intensity contrast ratio between the output logic states of 1 and 0 of 30 dB were realized simultaneously. This work not only opens up the possibility of realization of large-scale integrated information processing chips based on integrated plasmonic circuits, but also offers a way to overcome the intrinsic limitation of the serious losses of SPPs for on-chip integration applications.

## Methods

### Sample fabrication

300-nm-thick gold films were fabricated using a laser molecular beam epitaxy (LMBE) system (LMBE 450, SKY, China). We used the output beam (at an operating wavelength of 248 nm with a pulse repetition rate of 5 Hz) from an excimer laser system (COMPexPro 205, Coherent, USA) as the excitation light source. The beam was focused on a gold target mounted on a rotating holder that was located 15 mm from the silicon dioxide substrates. The typical energy density of the excitation laser was approximately 450 mJ/cm^2^. A focused ion-beam etching system (DB 235, FEI, USA) was used to prepare the U-shaped plasmonic waveguides, the XOR gates, and sample parity checkers.

### Nano-spectroscopy measurement setup

In the experiments, a nano-spectroscopy measurement system was used to measure both the waveguiding properties of the plasmonic waveguides and the logic performance of the sample all-optical logic parity checker. The input-coupling grating was normally illuminated from the rear using a home-made fiber laser system. The optically-thick gold film can prevent the direct transmission of the incident laser beam. The incident signal laser beam was focused into a 30-μm-diameter spot. The center of the focused laser beam was located at the midpoint of a line segment formed by input-coupling ports A and D of the sample all-optical logic parity checker, and at the midpoint of a line segment formed by input-coupling ports A and B of the sample all-optical logic XOR gate. This guarantees that all input-coupling ports can be excited uniformly, i.e., the incident signal intensities would have almost identical values for all input-coupling ports. The line width of the laser spectrum curve was less than 1.5 nm, thus ensuring that only the desired quasi-monochromatic SPPs can be excited by the input-coupling gratings. The guided SPP mode was scattered by a decoupling grating etched into the output port of the plasmonic waveguide. The scattered light was then collected using a long working distance objective (Mitutoyo 20, NA = 0.58), and imaged using a CCD imager. The intensity of the scattered light at the decoupling port was obtained directly from the CCD image, and no normalization was required.

## Additional Information

**How to cite this article**: Wang, F. *et al.* Nanoscale on-chip all-optical logic parity checker in integrated plasmonic circuits in optical communication range. *Sci. Rep.* **6**, 24433; doi: 10.1038/srep24433 (2016).

## Supplementary Material

Supplementary Information

## References

[b1] Kumar A. & Raghuwanshi S. K. Implementation of optical gray code converter and even parity checker using the electro-optic effect in the Mach-Zehnder interferometer. Opt. Quant. Electron. 47, 2117–2140 (2015).

[b2] Mehra R., Jaiswal S. & Dixit H. K. Parity checking and generating circuit with semiconductor optical amplifier in all-optical domain. Optik 124, 4744–4745 (2013).

[b3] Dimitriadou E., Zoiros K. E., Chattopadhyay T. & Roy J. N. Design of ultrafast all-optical 4-bit parity generator and checker using quantum-dot semiconductor optical amplifier-based Mach-Zehnder interferometer. J. Comput. Electron. 12, 481–489 (2013).

[b4] Bhattacharyya A., Gayen D. K. & Chattopadhyay T. All-optical parallel parity generator circuit with the help of semiconductor optical amplifier (SOA)-assisted Sagnac switches. Opt. Commun. 313, 99–105 (2014).

[b5] Srivastava V. K. & Priye V. All-optical 4-bit parity checker design. Optica Applicata 41, 157–164 (2011).

[b6] Djordjevic I. B. Photonic entanglement-assisted quantum low-density parity-check encoders and decoders. Opt. Lett. 35, 1464–1466 (2010). 20436604 10.1364/OL.35.001464

[b7] Djordjevic I. B., Cvijetic M., Xu L. & Wang T. Using LDPC-coded modulation and coherent detection for ultra highspeed optical transmission. J. Lightwave Technol. 25, 3619–3625 (2007).

[b8] Chowdhury K. R., De D. & Mukhopadhyay S. Parity checking and generating circuit with nonlinear material in all-optical domain. Chin. Phys. Lett. 22, 1433–1435 (2005).

[b9] Ghosh A. K. Parity generator and parity checker in the modified trinary number system using savart plate and spatial light modulator. Optoelectron. Lett. 6, 325–327 (2010).

[b10] Poustie A. J., Blow K. J., Kelly A. E. & Manning R. J. All-optical parity checker with bit-differential delay. Opt. Commun. 162, 37–43 (1999).

[b11] Oconnor D., Mccurry M., Lafferty B. & Zayats A. V. Plasmonic waveguide as an efficient transducer for high-density data storage. Appl. Phys. Lett. 95, 171112 (2009).

[b12] Kriesch A. *et al.* Functional plasmonic nanocircuits with low insertion and propagation losses. Nano Lett. 13, 4539–4545 (2013). 23962146 10.1021/nl402580c

[b13] Schuller J. A. *et al.* Plasmonics for extreme light concentration and manipulation. Nature Mater. 9, 193–204 (2010). 20168343 10.1038/nmat2630

[b14] Fu Y. L. *et al.* All-optical logic gates based on nanoscale plasmonic slot waveguides. Nano Lett. 12, 5784–5790 (2012). 23116455 10.1021/nl303095s

[b15] Johnson P. B. & Christy R. W. Optical constants of the noble metals. Phys. Rev. B 6, 4370 (1972).

[b16] Li X. E., Jiang T., Shen L. F. & Deng X. H. Subwavelength guiding of channel plasmon polaritons by textured metallic grooves at telecom wavelengths. Appl. Phys. Lett. 102, 031606 (2013).

[b17] Volkov V. S., Bozhevolnyi S. I., Devaux E. & Ebbesen T. W. Compact gradual bends for channel plasmon polaritons. Opt. Express 14, 4494–4503 (2006). 19516603 10.1364/oe.14.004494

[b18] Liu Y. *et al.* All optical logic gates based on two-dimensional low-refractive-index nonlinear photonic crystal slabs. Opt. Express 19, 1945–1953 (2011). 21369010 10.1364/OE.19.001945

[b19] Nozaki K. *et al.* Sub-femtojoule all-optical switching using a photonic-crystal nanocavity. Nature Photon. 4, 477–483 (2010).

[b20] Ishi T., Fujikata T., Makita K., Baba T. & Ohashi K. Si nanophotodiode with a surface plasmon antenna. Jpn. J. Appl. Phys. 44, 364–366 (2005).

[b21] Wood J. J., Lafone L., Hamm J. M., Hess O. & Oulton R. F. Plasmonic CROWs for tunable dispersion and high quality cavity modes. Sci. Rep. 5, 17724 (2015). 26631579 10.1038/srep17724 PMC4668557

[b22] Weeber J. C. *et al.* Surface-plasmon hopping along coupled coplanar cavities. Phys. Rev. B 76, 113405 (2007).

[b23] Balci S., Kocabas A., Kocabas C. & Aydinli A. Slowing surface plasmon polaritons on plasmonic coupled cavities by tuning grating grooves. Appl. Phys. Lett. 97, 131103 (2010).

