# Supplementary material for: Nanoscale on-chip all-optical logic parity checker in integrated plasmonic circuits in optical communication range
Source: Sci Rep. 2016 Apr 13;6:24433. doi: 10.1038/srep24433 (PMC4829911; doi:10.1038/srep24433)
Supplement: Supplementary Information [file srep24433-s1.pdf]

Supplementary Information

Nanoscale on-chip all-optical logic parity checker in integrated plasmonic circuits in optical communication range

Feifan Wang[1,*], Zibo Gong[1,*], Xiaoyong Hu[1,2], Xiaoyu Yang[1], Hong Yang[1] & Qihuang Gong[1,2]

[1] State Key Laboratory for Mesoscopic Physics & Department of Physics, Peking University, Beijing 100871, People's Republic of China

[2] Collaborative Innovation Center of Quantum Matter, Beijing 100871, People's Republic of China

*These authors contributed equally to this work.

1 More logic operation performance of all-optical logic parity checker

To discriminate the parity of the number of logic “1” for the incident four-bit logic signal “0001”, we etched one coupling grating connected with triangular air grooves in the input port of plasmonic waveguide D, as shown in Fig. S1(a1), which makes that the SPP mode can be excited in plasmonic waveguide D, and no SPP mode can be excited in the plasmonic waveguides A, B, and C. The measured CCD image under excitation of a 1560 nm CW laser is shown in Fig. S1(a2). There is a strong signal output from the decoupling grating of the output waveguide O with an intensity of 35 au. The outputs of the two XOR gates in the first level were “0” and “1”, respectively, which makes that the output of the XOR gate in the second level become “1”. This indicates that the incident four-bit logic signal “0001” has an odd parity of logic “1”. The calculated electric-field distribution of the all-optical parity checker under excitation of a 1560 nm CW laser is shown in Fig. S1(a3). Strong scattering signal can be obtained from the output waveguide O, which confirms the measured results.

To discriminate the parity of the number of logic “1” for the incident four-bit logic signal “0010”, we etched one coupling grating connected with triangular air grooves in the input port of plasmonic waveguide C, as shown in Fig. S1(b1), which makes that the SPP mode can be excited in plasmonic waveguide C, and no SPP mode can be excited in the plasmonic waveguides A, B, and D. The measured CCD image under excitation of a 1560 nm CW laser is shown in Fig. S1(b2). There is a strong signal output from the decoupling grating of the output waveguide O with an intensity of 34 au. The outputs of the two XOR gates in the first level were “0” and “1”, respectively, which makes that the output of the XOR gate in the second level become “1”. This indicates that the incident four-bit logic signal “0010” has an odd parity of

logic “1”. The calculated electric-field distribution of the all-optical parity checker under excitation of a 1560 nm CW laser is shown in Fig. S1(b3). Strong scattering signal can be obtained from the output waveguide O, which confirms the measured results.

To discriminate the parity of the number of logic “1” for the incident four-bit logic signal “0011”, we etched two coupling grating connected with triangular air grooves in the input port of plasmonic waveguide C and D, as shown in Fig. S1(c1), which makes that the SPP mode can be excited in plasmonic waveguide C and D, and no SPP mode can be excited in the plasmonic waveguides A and B. The measured CCD image under excitation of a 1560 nm CW laser is shown in Fig. S1(c2). There is a very weak signal output from the decoupling grating of the output waveguide O with an intensity of 0.005 au. Both the outputs of the two XOR gates in the first level was “0”, which makes that the output of the XOR gate in the second level become “0”. This indicates that the incident four-bit logic signal “0011” has an even parity of logic “1”. The calculated electric-field distribution of the all-optical parity checker under excitation of a 1560 nm CW laser is shown in Fig. S1(c3). No scattering signal can be obtained from the output waveguide O, which confirms the measured results.

To discriminate the parity of the number of logic “1” for the incident four-bit logic signal “0101”, we etched two coupling grating connected with triangular air grooves in the input port of plasmonic waveguide B and D, as shown in Fig. S1(d1), which makes that the SPP mode can be excited in plasmonic waveguide B and D, and no SPP mode can be excited in the plasmonic waveguides A and C. The measured CCD image under excitation of a 1560 nm CW laser is shown in Fig. S1(d2). There is a very weak signal output from the decoupling grating of the output waveguide O with an intensity of 0.004 au. Both the outputs of the two XOR gates in the first level was “1”, which makes that the output of the XOR gate in the second level become “0”.

This indicates that the incident four-bit logic signal "0101" has an even parity of logic "1". The calculated electric-field distribution of the all-optical parity checker under excitation of a 1560 nm CW laser is shown in Fig. S1(d3). No scattering signal can be obtained from the output waveguide O, which confirms the measured results.

To discriminate the parity of the number of logic "1" for the incident four-bit logic signal "0110", we etched two coupling grating connected with triangular air grooves in the input port of plasmonic waveguide B and C, as shown in Fig. S1(e1), which makes that the SPP mode can be excited in plasmonic waveguide B and C, and no SPP mode can be excited in the plasmonic waveguides A and D. The measured CCD image under excitation of a 1560 nm CW laser is shown in Fig. S1(e2). There is a very weak signal output from the decoupling grating of the output waveguide O with an intensity of 0.006 au. Both the outputs of the two XOR gates in the first level was "1", which makes that the output of the XOR gate in the second level become "0". This indicates that the incident four-bit logic signal "0110" has an even parity of logic "1". The calculated electric-field distribution of the all-optical parity checker under excitation of a 1560 nm CW laser is shown in Fig. S(e3). No scattering signal can be obtained from the output waveguide O, which confirms the measured results.

To discriminate the parity of the number of logic "1" for the incident four-bit logic signal "1000", we etched one coupling grating connected with triangular air grooves in the input port of plasmonic waveguide A, as shown in Fig. S1(f1), which makes that the SPP mode can be excited in plasmonic waveguide A, and no SPP mode can be excited in the plasmonic waveguides B, C and D. The measured CCD image under excitation of a 1560 nm CW laser is shown in Fig. S1(f2). There is a strong signal output from the decoupling grating of the output waveguide O with an intensity of 33 au. The outputs of the two XOR gates in the first level were

"0" and "1", respectively, which makes that the output of the XOR gate in the second level become "1". This indicates that the incident four-bit logic signal "1000" has an odd parity of logic "1". The calculated electric-field distribution of the all-optical parity checker under excitation of a 1560 nm CW laser is shown in Fig. S1(f3). Strong scattering signal can be obtained from the output waveguide O, which confirms the measured results.

To discriminate the parity of the number of logic "1" for the incident four-bit logic signal "1010", we etched two coupling grating connected with triangular air grooves in the input port of plasmonic waveguide A and C, as shown in Fig. S1(g1), which makes that the SPP mode can be excited in plasmonic waveguide A and C, and no SPP mode can be excited in the plasmonic waveguides B and D. The measured CCD image under excitation of a 1560 nm CW laser is shown in Fig. S1(g2). There is a very weak signal output from the decoupling grating of the output waveguide O with an intensity of 0.003 au. Both the outputs of the two XOR gates in the first level was "1", which makes that the output of the XOR gate in the second level become "0". This indicates that the incident four-bit logic signal "1010" has an even parity of logic "1". The calculated electric-field distribution of the all-optical parity checker under excitation of a 1560 nm CW laser is shown in Fig. S1(g3). No scattering signal can be obtained from the output waveguide O, which confirms the measured results.

To discriminate the parity of the number of logic "1" for the incident four-bit logic signal "1011", we etched three coupling grating connected with triangular air grooves in the input port of plasmonic waveguide A, C and D, as shown in Fig. S1(h1), which makes that the SPP mode can be excited in plasmonic waveguide A, C and D, and no SPP mode can be excited in the plasmonic waveguide B. The measured CCD image under excitation of a 1560 nm CW laser is shown in Fig. S1(h2). There is a strong signal output from the decoupling grating of the output

waveguide O with an intensity of 40 au. The outputs of the two XOR gates in the first level were "1" and "0", respectively, which makes that the output of the XOR gate in the second level become "1". This indicates that the incident four-bit logic signal "0010" has an odd parity of logic "1". The calculated electric-field distribution of the all-optical parity checker under excitation of a 1560 nm CW laser is shown in Fig. S1(h3). Strong scattering signal can be obtained from the output waveguide O, which confirms the measured results.

To discriminate the parity of the number of logic "1" for the incident four-bit logic signal "1100", we etched two coupling grating connected with triangular air grooves in the input port of plasmonic waveguide A and B, as shown in Fig. S1(i1), which makes that the SPP mode can be excited in plasmonic waveguide A and B, and no SPP mode can be excited in the plasmonic waveguides C and D. The measured CCD image under excitation of a 1560 nm CW laser is shown in Fig. S1(i2). There is a very weak signal output from the decoupling grating of the output waveguide O with an intensity of 0.005 au. Both the outputs of the two XOR gates in the first level was "0", which makes that the output of the XOR gate in the second level become "0". This indicates that the incident four-bit logic signal "1100" has an even parity of logic "1". The calculated electric-field distribution of the all-optical parity checker under excitation of a 1560 nm CW laser is shown in Fig. S1(i3). No scattering signal can be obtained from the output waveguide O, which confirms the measured results.

To discriminate the parity of the number of logic "1" for the incident four-bit logic signal "1101", we etched three coupling grating connected with triangular air grooves in the input port of plasmonic waveguide A, B and D, as shown in Fig. S1(j1), which makes that the SPP mode can be excited in plasmonic waveguide A, B and D, and no SPP mode can be excited in the plasmonic waveguide C. The measured CCD image under excitation of a 1560 nm CW laser is

shown in Fig. S1(j2). There is a strong signal output from the decoupling grating of the output waveguide O with an intensity of 38 au. The outputs of the two XOR gates in the first level were "0" and "1", respectively, which makes that the output of the XOR gate in the second level become "1". This indicates that the incident four-bit logic signal "0010" has an odd parity of logic "1". The calculated electric-field distribution of the all-optical parity checker under excitation of a 1560 nm CW laser is shown in Fig. S1(j3). Strong scattering signal can be obtained from the output waveguide O, which confirms the measured results.

To discriminate the parity of the number of logic "1" for the incident four-bit logic signal "1110", we etched three coupling grating connected with triangular air grooves in the input port of plasmonic waveguide A, B and C, as shown in Fig. S1(k1), which makes that the SPP mode can be excited in plasmonic waveguide A, B and C, and no SPP mode can be excited in the plasmonic waveguide B. The measured CCD image under excitation of a 1560 nm CW laser is shown in Fig. S1(k2). There is a strong signal output from the decoupling grating of the output waveguide O with an intensity of 38 au. The outputs of the two XOR gates in the first level were "0" and "1", respectively, which makes that the output of the XOR gate in the second level become "1". This indicates that the incident four-bit logic signal "1110" has an odd parity of logic "1". The calculated electric-field distribution of the all-optical parity checker under excitation of a 1560 nm CW laser is shown in Fig. S1(k3). Strong scattering signal can be obtained from the output waveguide O, which confirms the measured results.

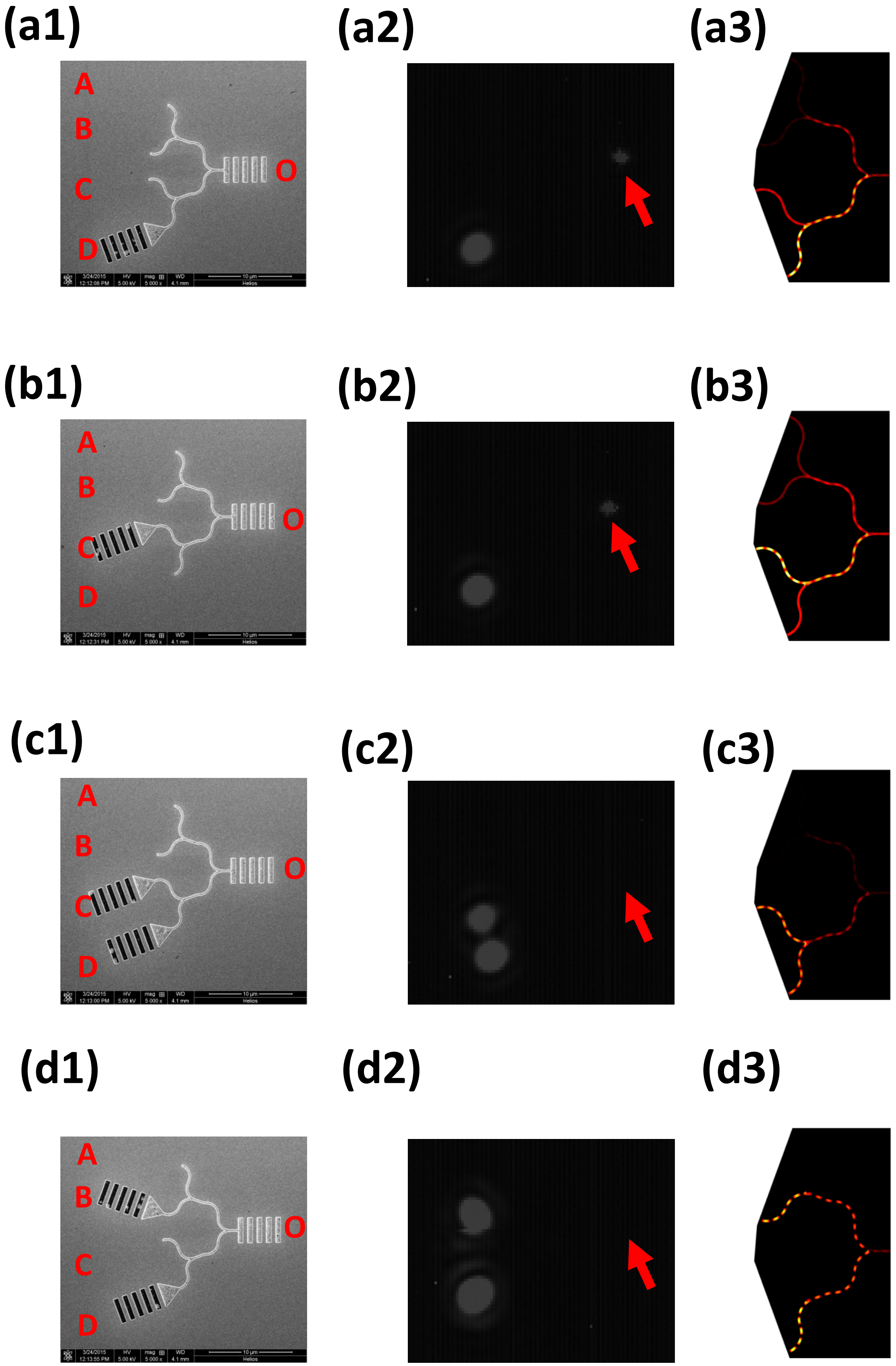
(a1)
(a2)
(a3)
(b1)
(b2)
(b3)
(c1)
(c2)
(c3)
(d1)
(d2)
(d3)
A
B
C
D
O

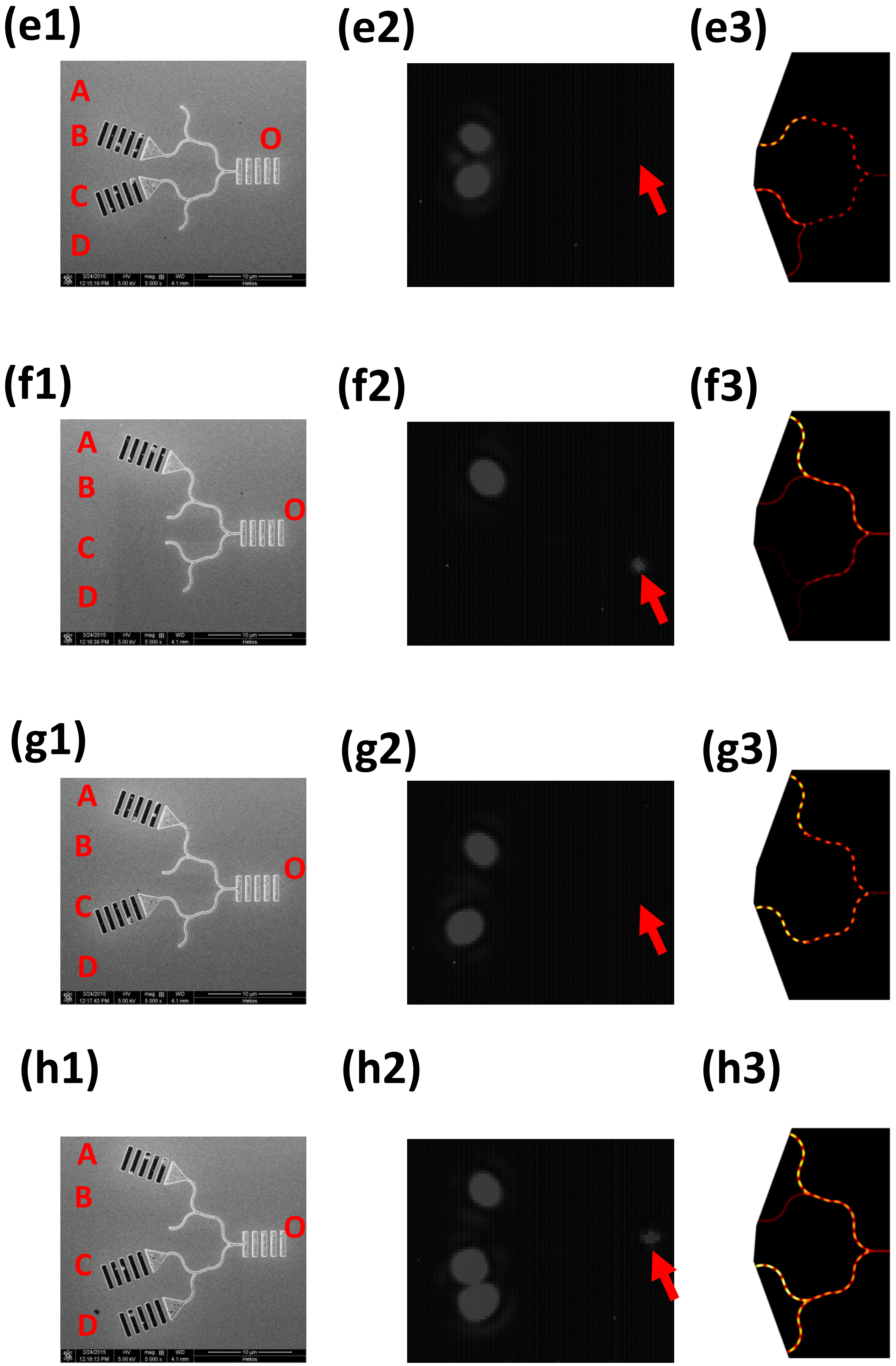
(e1)
(e2)
(e3)
A
B
C
D
O
(f1)
(f2)
(f3)
A
B
C
D
O
(g1)
(g2)
(g3)
A
B
C
D
O
(h1)
(h2)
(h3)
A
B
C
D
O

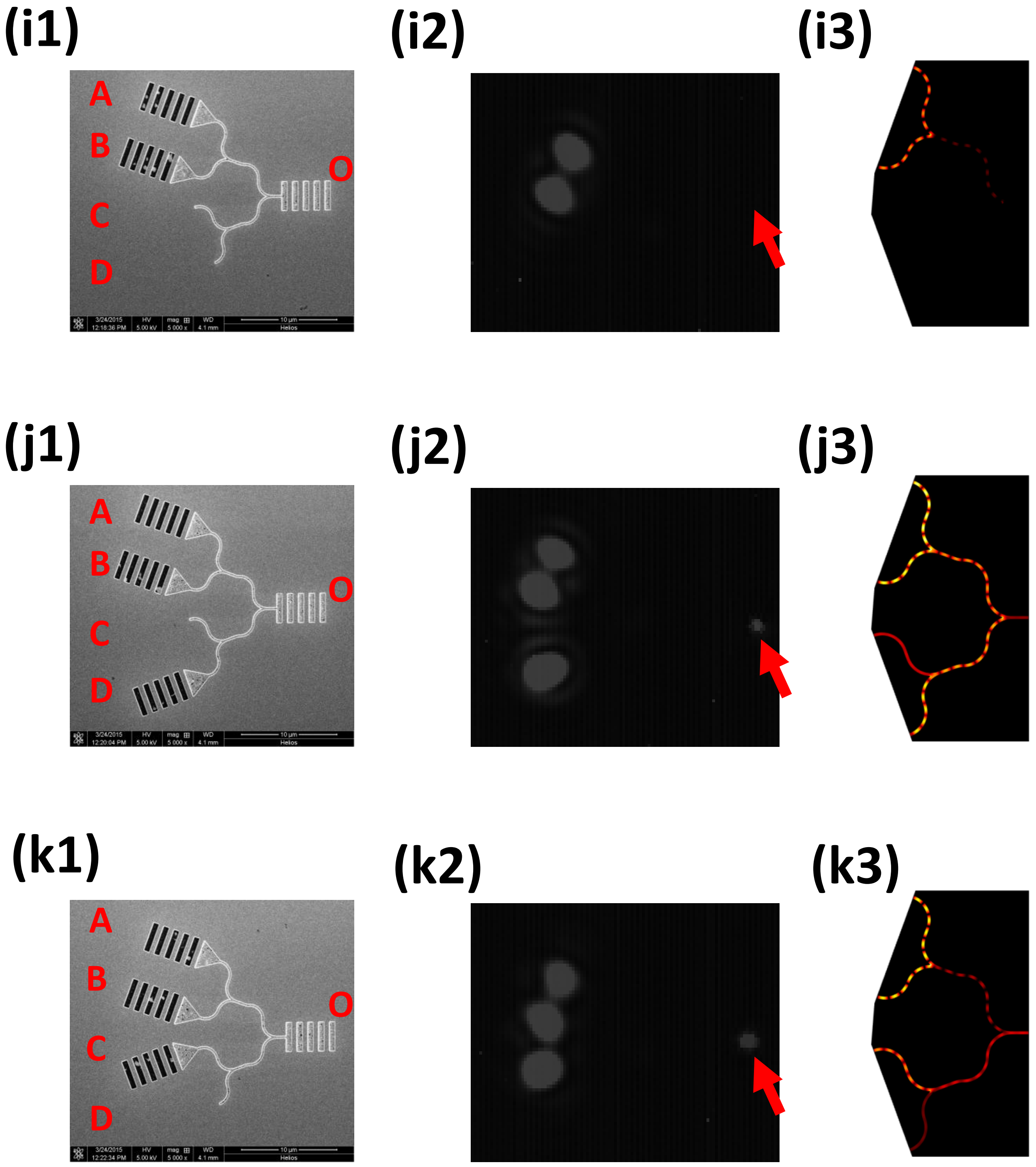

Figure S1 | Logic operation of the of All-optical logic parity checker for different incident logic signals. The SEM image of the sample (a1), measured CCD image under excitation of a 1560 nm CW laser (a2), and simulated results electric-field distribution for a 1560 nm CW incident laser (a3) for the incident logic signal “0001”. The SEM image of the

sample (b1), measured CCD image under excitation of a 1560 nm CW laser (b2), and simulated results electric-field distribution for a 1560 nm CW incident laser (b3) for the incident logic signal "0010". The SEM image of the sample (c1), measured CCD image under excitation of a 1560 nm CW laser (c2), and simulated results electric-field distribution for a 1560 nm CW incident laser (c3) for the incident logic signal "0011". The SEM image of the sample (d1), measured CCD image under excitation of a 1560 nm CW laser (d2), and simulated results electric-field distribution for a 1560 nm CW incident laser (d3) for the incident logic signal "0101". The SEM image of the sample (e1), measured CCD image under excitation of a 1560 nm CW laser (e2), and simulated results electric-field distribution for a 1560 nm CW incident laser (e3) for the incident logic signal "0110". The SEM image of the sample (f1), measured CCD image under excitation of a 1560 nm CW laser (f2), and simulated results electric-field distribution for a 1560 nm CW incident laser (f3) for the incident logic signal "1000". The SEM image of the sample (g1), measured CCD image under excitation of a 1560 nm CW laser (g2), and simulated results electric-field distribution for a 1560 nm CW incident laser (g3) for the incident logic signal "1010". The SEM image of the sample (h1), measured CCD image under excitation of a 1560 nm CW laser (h2), and simulated results electric-field distribution for a 1560 nm CW incident laser (h3) for the incident logic signal "1011". The SEM image of the sample (i1), measured CCD image under excitation of a 1560 nm CW laser (i2), and simulated results electric-field distribution for a 1560 nm CW incident laser (i3) for the incident logic signal "1100". The SEM image of the sample (j1), measured CCD image under excitation of a 1560 nm CW laser (j2), and simulated results electric-field distribution for a 1560 nm CW incident laser (j3) for the incident logic signal "1101". The SEM image of the sample (k1), measured CCD image under excitation of a 1560 nm CW laser (k2), and simulated results electric-field

distribution for a 1560 nm CW incident laser (k3) for the incident logic signal “1110”. Arrow indicates the position of the decoupling grating of the output waveguide O.

2 Difference of this manuscript from Ref. [14] [Nano Letters 12, 5784 (2012)]

The main differences of this manuscript from Ref. [14] [Nano Letters 12, 5784 (2012)]lies in the following factors:

(1) The purpose is completely different. The Ref. [14] [Nano Letters 12, 5784 (2012)] aims at realizing all-optical XNOR, XOR, NOT, and OR logic gates using plasmonic sot waveguides, operating at the wavelength of 830 nm. Only four simple unit logic gates were realized in Ref. [14] [Nano Letters 12, 5784 (2012)]. Our manuscript aims at realizing a nanoscale chip-integrated all-optical logic parity checker in integrated plasmonic circuits directly in the optical communication range. The all-optical logic parity checker is a kind of complex logic devices, which are composed of several simple unit logic gates, and can perform complicated logic operation functions that the single simple unit logic gate can not fulfill.

(2) The configuration of the sample is completely different. In Ref. [14] [Nano Letters 12, 5784 (2012)], the all-optical XNOR, XOR, NOT, and OR logic gates consist of two or three input plasmonic sot waveguides and one output plasmonic sot waveguide. In our manuscript, the all-optical logic parity checker consists of two-level cascaded all-optical XOR logic gates realized using U-shaped plasmonic waveguide. The sample configuration of our manuscript is very complicated, and completely different from that of the Ref. [14] [Nano Letters 12, 5784 (2012)].

(3) The extensibility of the concept is completely different. In Ref. [14] [Nano Letters 12, 5784 (2012)], the propagation losses are relatively large for the plasmonic slot waveguide in the near-infrared range because of relatively large intrinsic ohmic losses of gold in the near-infrared range. The propagation length of surface plasmon polariton modes in the

plasmonic slot waveguides is only 10 μm in the near-infrared range, which means that it is not possible to realize the complicated and cascaded all-optical logic devices based on the physical concept of Ref. [14] [Nano Letters 12, 5784 (2012)]. In our manuscript, the propagation losses are relatively small for the U-shaped plasmonic waveguides in the optical communication range because of relatively small intrinsic ohmic losses of gold in the optical communication range. The propagation length of surface plasmon polariton modes in the U-shaped plasmonic waveguides is about 41 μm in the optical communication range, which is confirmed by the calculations of Oconnor *et al*[1]. This indicates that it is suitable to realize the complicated and cascaded all-optical logic devices based on the physical concept of our manuscript.

Therefore, our manuscript is completely different from Ref. [14] [Nano Letters 12, 5784 (2012)].

3 More discussion of U-shaped plasmonic waveguides

For the U-shaped plasmonic waveguides used in our experiment, the maximum intensity of the power density profile of the guided SPP modes is located at the gold-air interface around two vertexes in the upper side, as shown in Fig. 1(c), which is confirmed by the calculated results of Li *et al*[2]. Therefore, the influences of the roughness of two lateral gold walls of the U-shaped plasmonic waveguide on the propagation losses of U-shaped plasmonic waveguides can be neglected, which also indicates that U-shaped plasmonic waveguides possess high tolerance for structural imperfections. According to our calculation, the propagation length of the U-shaped plasmonic waveguides used in our experiment was 41 μm in the optical communication range, which is confirmed by the calculations of Oconnor *et al*[1]. Kriesch *et al.* pointed out that the propagation length of the conventional plasmonic slot waveguides was only 34 μm in the optical communication range[3]. A part of the reason lies in that the maximum intensity of the power density profile of the guided SPP modes is located at the gold-air interface of the slot region for the conventional plasmonic slot waveguides, which means that the roughness of two lateral gold walls of the conventional plasmonic slot waveguide seriously influence propagation losses[3]. Jiang *et al.* also pointed out that the U-shaped plasmonic waveguides had stronger light-field localization effect and higher transmission through sharp bends compared with conventional pllasmonic slot waveguides because of different waveguide configuration and field distribution of guided SPP modes[4].

4 Discussion of Influences of impact fabrication on SPP interference

Lipp *et al.* have pointed out that two lateral walls of the slot waveguide in a thick gold film were oblique, because of the Gaussian wings of the focused Ga+ ion beam of the FIB etching system[5]. For sample used in our experiment, the depth of the air groove was only 100 nm for the U-shaped plasmonic waveguide, with the air groove width of 200 nm in the upper side and 195 nm in the bottom. There is a small difference between the fabricated sample and the designed model. Moreover, because the maximum intensity of the power density profile of the guided SPP modes is located at the gold-air interface around two vertexes in the upper side of the U-shaped plasmonic waveguide, the influences oblique lateral wall of the waveguide on the interference properties of waveguides A and B can be neglected. So, good agreement was obtained in the phase and amplitude between experimental and simulated interference pattern, as shown in Figs. 2 and 3 of the manuscript.

For a plasmonic slot waveguide etched through a 300-nm-thick gold film, the slot width was 200 nm in the upper side and 180 nm in the bottom according to our measurement. The wavelength of the guided SPP mode of the plasmonic slot waveguide was 1000 nm. So if a optical path difference of 500nm is designed between waveguide A and B, a perfect destructive interference can be obtained in the output waveguide for the guided SPP modes propagating plasmonic waveguides A and B under excitation of a 1560 nm CW signal laser beam.

References

1. Oconnor, D., Mccurry, M., Lafferty, B. & Zayats, A. V. Plasmonic waveguide as an efficient transducer for high-density data storage. *Appl. Phys. Lett.* **95**, 171112 (2009).
2. Li, X. E., Jiang, T., Shen, L. F. & Deng, X. H. Subwavelength guiding of channel plasmon polaritons by textured metallic grooves at telecom wavelengths. *Appl. Phys. Lett.* **102**, 031606 (2013).
3. Kriesch, A. *et al.* Functional plasmonic nanocircuits with low insertion and propagation losses. *Nano Lett.* **13**, 4539-4545 (2013).
4. Jiang, T. *et al.* Realization of tightly confined channel plasmon polaritons at low frequencies. *Appl. Phys. Lett.* **99**, 261103 (2011).
5. Lipp, S. *et al.* Investigations on the topology of structures milled and etched by focused ion beams. *J. Vac. Sci. Technol. B* **14**, 3996-3999 (1996).
